# Biomechanical Comparison of Two Different Sutures for the Tensile Strength of the Pullout Repair of Posterior Meniscal Root Tear

**DOI:** 10.7759/cureus.42378

**Published:** 2023-07-24

**Authors:** Tsuneari Takahashi, Katsushi Takeshita

**Affiliations:** 1 Department of Orthopaedic Surgery, Ishibashi General Hospital, Shimotsuke, JPN; 2 Department of Orthopaedics, Jichi Medical University, Shimotsuke, JPN

**Keywords:** transtibial pull-out repair, porcine model, tape, surgery, medial meniscus posterior root tear

## Abstract

Background

Medial meniscal posterior root tear has garnered attention because it causes increased joint contact pressure and loss of load distribution function. Currently, the common treatment for posterior meniscal root tears is transtibial pullout repair, and loop suture remains the gold standard procedure.

Purpose

The aim of this study is to validate whether the meniscus-suture complex exhibits better structural properties after loop stitch using a suture tape versus the conventional thread.

Study design and methods

This is a controlled laboratory study. In total, 20 menisci harvested from castrated male pigs were prepared and randomly assigned to two suturing techniques: loop stitch using ULTRATAPE (n = 10, tape group) or loop stitch using 2-0 UltraBraid (n = 10, control group). The single-loop stitch meniscus-suture technique was used. A single suture using ULTRATAPE or 2-0 UltraBraid was placed through the meniscus 5 mm medial from the resected edge of the posterior meniscus horn. The meniscus-suture complex specimens were mounted on a tensile tester to apply tensile load on the meniscus-suture complex parallel to the long axis of the stitched suture materials. Each specimen was stretched to failure at a crosshead speed of 50 mm/minute. The structural properties of the meniscus-suture complex (maximum load, linear stiffness, and elongation at failure) were determined.

Results

In the tape group, all the repaired complexes were torn at the ligature; conversely, in the control group, the threads were torn in three of 10 specimens, and the repaired complexes in the remaining seven specimens were torn at the ligature. No significant between-group differences were observed in linear stiffness (9.0 ± 4.1 vs 7.1 ± 2.9 N/mm) and elongation at failure (15.8 ± 6.1 vs 19.6 ± 20.1 mm); however, the tape group exhibited a significantly higher average maximum load than the control group (104.6 ± 41.2 vs 62.6 ± 32.1 N; *P *= 0.020).

Conclusion

Suture tape can be a potential alternative to the conventional thread for Medial meniscal posterior root tear repair.

Clinical relevance

Medial meniscal posterior root tear repair using tape rather than the conventional thread may be beneficial in obtaining better structural properties.

## Introduction

Menisci are soft tissues that play an important role in load distribution and joint stability [1]. The hoop shape of menisci is crucial in maintaining their function. Meniscal tear, which is caused by the combination of degeneration and excessive mechanical loading, is a common finding in knee surgery [2]. Recently, medial meniscal posterior root tear (MMPRT) has been garnering attention as it causes a significant increase in joint contact pressure and loss of load distribution function, thereby resulting in a rapid progression of knee osteoarthritis or knee osteonecrosis [3]. To restore meniscal function, anatomical reduction of the displacement is considered a prerequisite, and a complete healing of the attachment of the meniscal root is required to maintain meniscal function. The common treatment for MMPRT is transtibial pullout repair.

However, early postoperative re-displacement of the torn meniscus that was repaired may lead to incomplete healing or healing in a non-anatomical position that alters the biomechanics of joint contact as observed in a non-anatomical root repair [4]. One of the main contributions to postoperative re-displacements was reported due to the meniscus-suture (MS) complex [5]. Regarding tensile strength, loop stitches commonly used in clinical settings are significantly inferior to simple stitches and the modified Mason-Allen suture in terms of the maximum strength and inferior to the latter in terms of the stiffness of the MS complex [6]. Even though more complex suture configurations are supposed to provide superior biomechanical properties, realistically, it is slightly difficult to apply them arthroscopically. Currently, the repair of a torn posterior meniscal root using loop suture remains the gold standard procedure, with the double-locking loop suture repair technique being associated with a significantly higher failure load than the single-locking loop suture repair technique [7]. Therefore, an alternative method is required to enhance the mechanical properties of the MS complex and to simplify the surgical procedure. To reduce displacements, the use of suture tape instead of the conventional thread has been proposed to increase the contact area at the tissue insertion points [5]. However, whether the MS complex exhibits better structural properties with the use of suture tape rather than the conventional thread remains controversial [8].

 Our current literature search showed that only a few studies have compared the structural properties of the MS complex using suture tape and the conventional thread.

We hypothesized that the MS complex exhibits better structural properties after loop stitch using suture tape compared with using the conventional thread. Thus, the present study aimed to validate this hypothesis.

## Materials and methods

Study design

A total of 20 menisci harvested from castrated male pigs aged four months (average weight: 36.4 ± 3.2 kg; weight range: 33.0-40.5 kg) that were originally purchased for another animal model study were prepared for biomechanical evaluation. No macroscopic degenerative or traumatic changes were observed in all the menisci, and these were immediately harvested and stored at −20°C. Then, they were thawed overnight at 4°C prior to testing and randomly assigned to two suturing techniques frequently used for transtibial pullout repair of MMPRT: loop stitch using ULTRATAPE (tape group, n = 10; Smith & Nephew Endoscopy, Andover, MA) or loop stitch using 2-0 UltraBraid (control group, n = 10; Smith & Nephew Endoscopy). All animal experiments were conducted in accordance with the rules and regulations of the Animal Care and Use Committee at our institution.

Surgical procedure for posterior meniscal root repair

**Figure 1:**
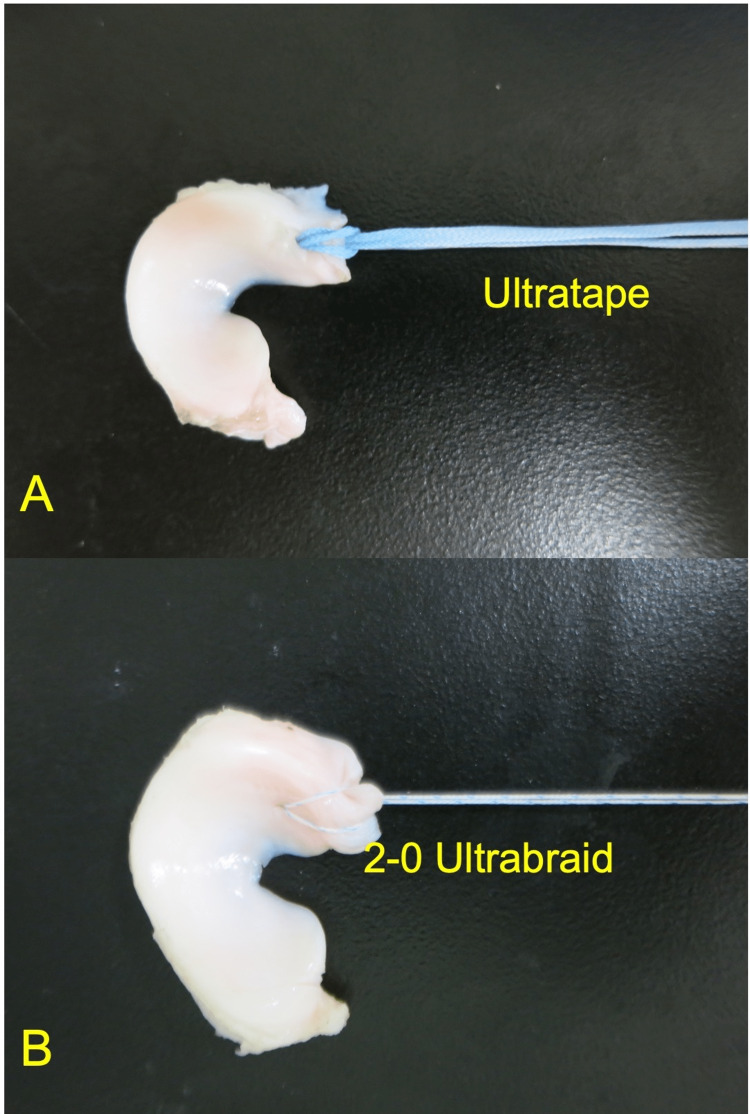
The single suture using ULTRATAPE (A) or 2-0 UltraBraid (B) was placed through the meniscus 5 mm medial from the resected edge of the posterior meniscus horn.

**Figure 2:**
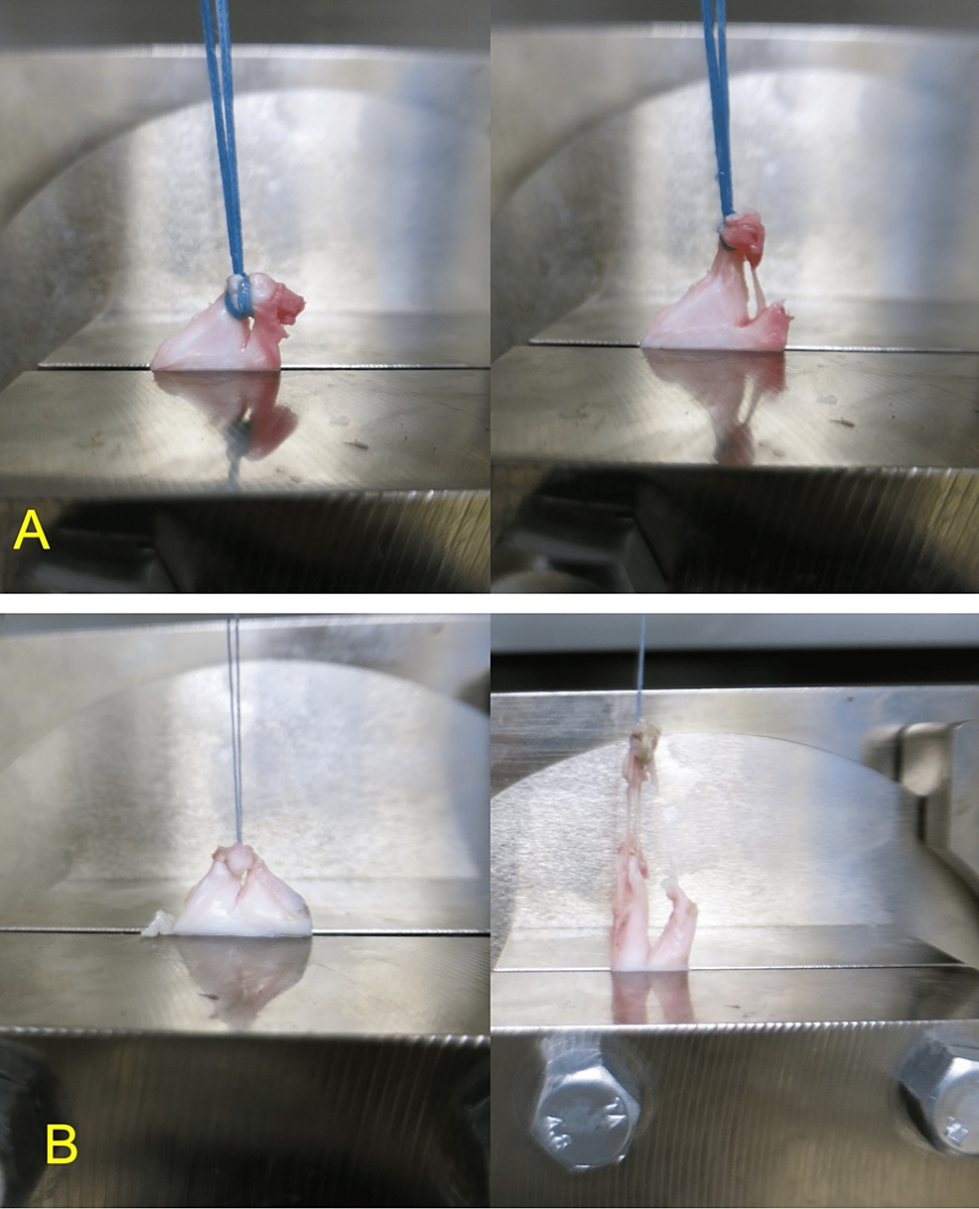
The failure mode of the repaired complexes in the tape group (A) and control group (B) at the time of tensile testing.

The single-loop stitch MS technique was used by one senior orthopedic surgeon (T.T.). FIRSTPASS MINI (Smith & Nephew Endoscopy) was used to place the loop suture. A single suture using ULTRATAPE (Figure 1A) or 2-0 UltraBraid (Figure 1B) was placed through the meniscus 5 mm medial from the resected edge of the posterior meniscus horn [9].

Structural properties of the MS complex

The prepared MS complex specimens were mounted on a tensile tester (TENSILON RTG-1250; Orientec Co., Ltd., Tokyo, Japan) with a set of specially designed grips to apply a tensile load to the MS complex parallel to the long axis of the stitched suture materials. This measurement system was similar to that used in previous biomechanical studies using large animals. Before testing, the specimen was preconditioned with a static preload of 5 N for 10 minutes, followed by 10 cycles of loading and unloading (3% strain) with a crosshead speed of 20 mm/minute. Following this, each specimen was stretched to failure using the same conditions with preconditioning at a crosshead speed of 50 mm/minute. These measurement conditions have been frequently used in previous studies with a large animal model [10]. Failure modes were video-recorded. A load-elongation curve was created using specific software (TENSILON Advanced Controller for Testing, Orientec Co., Ltd.). All tests were performed at room temperature, and the menisci were continuously kept moist using saline solution. The endpoint was the ultimate suture failure load. The structural properties of the MS complex (maximum load, linear stiffness, and elongation at failure) were determined from calculations obtained using a software.

Statistical analyses

A priori power analysis was performed using G* Power 3.1 (Franz Paul, Kiel, Germany) [11]. The sample size was calculated with 80% power with an effect size of 1.3 to test the study hypothesis. All data from statistical analyses were presented as mean ± standard deviation. Student’s t-test was used to evaluate between-group differences. All statistical analyses were performed using EZR software (http://www.jichi.ac.jp/saitama-sct/SaitamaHP.files/statmed.html) [12]. A p-value of <0.05 was considered significant.

## Results

Biomechanical evaluations of the MS complex

Regarding the failure mode in tensile testing, all the repaired complexes in the tape group were torn at the ligature (Figures 2A, 2B). Conversely, in the control group, the threads were torn in three of 10 specimens, and the repaired complexes in the remaining seven specimens were torn at the ligature). No significant between-group differences were observed in terms of linear stiffness (9.0 ± 4.1 vs 7.1 ± 2.9 N/mm) and elongation at failure (15.8 ± 6.1 vs 19.6 ± 20.1 mm); however, the tape group exhibited a significantly higher average maximum load than the control group (104.6 ± 41.2 vs 62.6 ± 32.1 N; P = 0.020) (Table 1).

**Table 1:** Outcomes of tensile testing

Parameters		Tape group (n = 10)	Control group (n = 10)	P-value
Maximum load (N)		104.6 (41.2)	62.6 (32.1)	0.0204
Linear stiffness (N/mm)		9.0 (4.1)	7.1 (2.9)	0.247
Elongation at failure (mm)		15.8 (6.1)	19.6 (20.1)	0.574

## Discussion

The present study clarified the biomechanical differences on the basis of tensile testing between MS complex specimens repaired using suture tape or the conventional thread. In tensile testing, the failure mode of the MS complex showed tearing of the ligature, not thread tearing, in all specimens in the tape group. However, the failure mode in the control group was different, and thread tearing was observed in 30% of the specimens. Regarding structural properties, no significant between-group differences were observed in terms of linear stiffness and elongation at failure. However, the tape group exhibited a significantly higher maximum load than the control group, and this finding was in accordance with that of the study of Cerminara et al., who showed that the MS interface should be the primary target of future optimization studies aimed at eliminating the displacement of transtibial pullout repair [5]. Even though the cycling loading test was not performed in this study, a greater contact area when the tape, rather than the thread, was used at the tissue insertion points might have contributed to the enhancement of the structural properties; furthermore, during the tensile testing, tape tearing was not observed, whereas the thread was torn in 30% of specimens. Because we cannot enhance the structural property of the injured posterior root of the medial meniscus, it is quite important to enhance the structural properties of the MS complex. The results of the current study show that the use of suture tape rather than the conventional thread may provide better structural properties to the MS complex. The average maximum load in the control group using the conventional 2-0 thread was 62.6 N, which was comparable with the average maximum load in tensile testing using human cadaveric specimens in the study by Stärke et al. [13]. Meanwhile, the maximum load in the tape group had increased to 104.6 N; even though the maximum load was significantly higher in this group than in the control group, it remained lower than the previously reported value of 359 N in the native human medial meniscus posterior root [14]. In that study, although the locking loop stitch was significantly stronger than the single suture, it yielded a lower maximum load than that in the native human medial meniscus posterior root. On the basis of the results of the present study, early excessive weight-bearing or range-of-motion exercise is not recommended even though the MS complex exhibited better structural properties when suture tape was used. Novel therapeutic strategies that enhance the meniscal healing process by influencing not only biomechanical factors related to the repair but also biological factors intrinsic to the meniscus are required.

The strength of our study was that the structural properties of porcine menisci might have been homogenous because of the controlled age at the time of euthanasia. However, the present study also had several limitations. First, porcine menisci were used in this study; thus, some of the structural and histological findings may not be directly applicable to the clinical practice for human menisci. Second, a limited number of specimens and implants were available for use, which lowered the available sample size in each group. Third, the average age of the pigs at the time of euthanasia was four months, and no obvious degenerations were observed in all menisci; moreover, castrated male pigs were used in this study. MMPRT is common in middle-aged women, and aging and female sex are important factors associated with meniscal degeneration [15]. Therefore, these results cannot be directly applied to the clinical practice for the menisci in middle-aged humans. However, in reality, it is difficult to obtain the menisci of middle-aged pigs in our experimental environment. Fourth, cyclic loading, which can help determine displacement, was not performed in this study. Thus, it should be performed in future studies. Fifth, the MS complex comprised only intact posterior root and suturing material. The influence of traction force to the posterior root of the medial meniscus at the time of injury, which is frequently observed in clinical settings, was not considered in this study. The forces acting on the posterior root of the medial meniscus may deteriorate the structural properties of the torn tissues. Therefore, whether the results of this study are applicable to clinical settings remains unclear. Thus, an in vivo biomechanical study using a large animal model with a certain follow-up period must be conducted to validate the effect of suture tape for the surgical treatment of MMPRT, and this will be the focus of our future study. Despite these limitations, the present study results provide valuable information on the efficacy of MMPRT repair using suture tape. Further specific studies must be conducted to overcome the limitations of the present study. In addition, clinical trials for this surgical procedure must be conducted to validate its cost-effectiveness, better clinical outcomes, and the conclusions of this study.

## Conclusions

MMPRT repair using tape rather than the conventional thread may be beneficial in obtaining better structural properties. Suture tape can be a potential alternative to the conventional thread for MMPRT.
